# Prebiotic Activity of Polysaccharides Extracted from *Gigantochloa Levis* (Buluh beting) Shoots

**DOI:** 10.3390/molecules17021635

**Published:** 2012-02-07

**Authors:** Aida Firdaus Muhammad Nurul Azmi, Shuhaimi Mustafa, Dzulkifly Md. Hashim, Yazid Abdul Manap

**Affiliations:** 1 Department of Microbiology, Faculty Biotechnology and Biomolecular Sciences, Universiti Putra Malaysia, 43400 Serdang, Selangor Darul Ehsan, Malaysia; 2 Department of Food Technology, Faculty of Applied Sciences, Universiti Teknologi MARA, 40450 Shah Alam, Selangor, Malaysia; 3 Halal Products Research Institute, Universiti Putra Malaysia, Putra Infoport, 43400 Serdang Selangor, Malaysia; 4 Department of Food Technology, Faculty of Food Science and Technology, Universiti Putra Malaysia, 43400 Serdang, Selangor Darul Ehsan, Malaysia

**Keywords:** polysaccharides, FOS, prebiotics, probiotics, optical density

## Abstract

Bamboo shoot crude polysaccharides (BSCP) extracted from the shoots of *Gigantochloa levis* gave about 3.27 ± 0.18% on dry basis and a very minute percentage of protein (0.02 ± 0.01%). The molecular weight of BSCP estimated by gel chromatography was found to be around 7.49 × 10^3^ Da, while the molecular weights of purified fractions (F1 to F5) were around 1550.96, 1471.63, 1685.78, 1691.61 and 1551.67 Da, respectively. The FTIR spectrum of BSCP revealed the possibility that the extract contains β-glucan, which can be considered a valuable compound for the medical and food industries. These relate to the resistance of BSCP towards artificial human gastric juice which is more than 99%. Prebiotic activity tested using BSCP as a carbon source showed significant increase in the growth of *B. animalis* ATCC 1053, *B. longum* BB 536 and *L. acidophilus* ATCC 4356 as compared to the use of FOS. Survivality of *S. choleraesuis* JCM 6977 was found to be slower in both BSCP and FOS. Study conducted reflects a good sign for the BSCP to be exploited as a promising prebiotic.

## 1. Introduction

Bamboo belongs to the Gramineae Bambusoideae and is widely distributed in China, Japan and other South-East Asian countries. It is a fast growing forestry plant and be regarded as a multipurpose plant as it was used widely in construction materials, furniture, papers, chopsticks, as well as a food source [1]. Panee [2] quoted a brief description by Geil over a century ago in his book “*A Yankee on the Yangtze*”, that reflects the usefulness of bamboo and the close relationship between bamboo and the daily life of Chinese people; “A man can sit in a bamboo house under a bamboo roof, on a bamboo chair at a bamboo table, with a bamboo hat on his head and bamboo sandals on his feet. He can at the same time hold in one hand a bamboo bowl, in the other hand bamboo chopsticks and eat bamboo sprouts. When through with his meal, which has been cooked over a bamboo fire, the table may be washed with a bamboo cloth, and he can fan himself with a bamboo suspension bridge, drink water from a bamboo ladle, and scrape himself with a bamboo scraper (handkerchief)”. Interestingly, no medical aspects are stressed in the description, even though bamboo has long been used in traditional Chinese and Asian medicine, especially in treating cold, hypertension, arteriosclerosis, cardiovascular disease as well as cancer. 

Nowadays, there is an increasing trend of consumer awareness towards the demand for functional foods, which are claimed to enhance the health of the consumer. Apart from other food ingredients, prebiotics are among those which have atttracted much attention recently [3]. The world demand for prebiotics is estimated to be around 167,700 tons and to be worth 390 million Euro [4]. A prebiotics is “a non-digestible and selectively fermented ingredient that allow specific changes, both in the composition and/or activity in the gastrointestinal microbiota that confers benefits upon host well-being and health” [5]. They cannot be digested by α-amylase or other hydrolases in the upper gut section of the intestinal tract [6]. In general, prebiotics can be considered as a ‘food’ for probiotics. Probiotics can be defined as “live microbial food supplements which benefit the health of consumers by maintaining or improving their intestinal microbial balance” [7].

Human intestinal microbiota is composed of more than 400 bacterial species, which make it a complex bacterial ecosystem. Bifidobacteria and lactobacilli are predominant members of the gut microbiota, with Bifidobacteria make up about 25% of the total number of bacteria present. They are best known for their beneficial and health promoting properties [8]. This allows Bifidobacterium and Lactobacillus genera being the most important probiotic strains for human use [9].

Many symbiotic relationships between probiotics and prebiotics have been studied in order to maximize their beneficial effects. Both bifidobacteria and lactobacilli were best known to utilize prebiotics in the GI tract, based on the fact that they contain relatively high amount of β-fructosidase and glycotransferases, respectivelys which enable them to break down polymers (the prebiotic) into smaller units and make it available as a substrate during fermentation [10,11]. Organic acids will be produced by lactic acid bacteria as a result of fermentation, thus providing an acidic environment in the colon which indirectly suppresses the growth of pathogens. This mechanism allows prebiotics to manipulate the composition of colonic microbiota in human gut [12], thus improving the host health [13] in return. These include enhancement of immune function, improve digestion and elimination of faeces as well as reducing the potential of getting irresistible bowel syndrome (IBS) [14].

Many studies have now confirmed that the prebiotics incorporated in the diet are a valid approach to the dietary manipulation of the colonic microbiota. This concept has gained global attention and is being manipulated for human health purposes. Because of increasing demand for prebiotics, there is a need to find a new source of prebiotics which is relatively low price as compared to commercially available prebiotics. Extensive studies are now focused on the prebiotic potential of polysaccharides extracted from natural sources such as mushroom (*Pleurotusostreatus* and *Pleurotuseryngii*) [15], rhizome of *Arthropodium cirratum* (Regarenga lily extract) [16], pitaya (dragon fruit) flesh [17], cashew apple (*Anacardium occidentale* L) juice [18], Bengal gram husk (*Cicerarietinum L*.) and wheat bran (*Triticum aestivum*) [19].

Apart from their unique taste and crispy texture, bamboo shoots were also reported to be highly nutritious. They contain a high percentage of dietary fiber, which includes hemicellulose, cellulose, pectin and lignin. These compounds gave about 8% of soluble fiber and 92% of insoluble fiber [20], which would help in lowering serum cholesterol and preventing cardiovascular disease [21,22]. They were also reported to have antioxidant activity, which may involve scavenging of free radicals [23]. Excessive consumption of bamboo shoots was however claimed to cause intestinal discomfort like bloating and flatulence. This is actually associated with the dietary content of bamboo shoots which served as a substrate for fermentation of intestinal bacterial. These phenomena are similar to that of consumption of prebiotics. These exposed another potential use of bamboo shoots as a source of prebiotics. Therefore, this study was conducted to reveal other medicinal usages of bamboo shoots as a prebiotic. The objectives of this study are: (i) to quantify the water soluble extract from the shoots of *Gigantochloa levis*; (ii) to characterize the extract obtained and (iii) to elucidate the function of extract in supporting the growth of *B. animalis*, *B. longum*, *L. acidophilus* and also its ability to suppress the growth of *Salmonella* spp.

## 2. Results and Discussion

### 2.1. Extraction of Crude Polysaccharides

Proper isolation and purification of polysaccharides from its original source will help in its identification and characterization. Prior removal of fat from the raw materials is necessary because fat can limit water penetration and thus affect the extraction efficiency. Lipid substances can be defatted using polar solvents such as chloroform-methanol solutions (95:5 v/v), ethanol (90% v/v) or dioxane and hexane [24]. Defatted samples were only then used for extraction.

Extraction of polysaccharides from plants is usually carried employing hot water. However, the yield of extracted polysaccharides depends on several factors such as temperature, time, ratio of water to raw materials used, as well as extraction number. Cai *et al.* [25] studied these effects. They reported that, extraction of polysaccharides at higher temperatures (more than 80 °C) will lead to hydrolysis of polysaccharides, thus reducing the extract yield. Lengthening the time of extraction was noted to increase the yield of polysaccharides to a certain extent, but excessive extraction time (more than 4 h) will induced changes in the molecular structures of polysaccharides, thus decreasing its extraction yield.

The ratio of water to raw materials used also influences the extraction yield. It was found that ratios of water to raw materials within the 3–4 range led to higher extraction yields, while adding more water resulted in no significant increment in the yield obtained. The number of extractions suggested was only twice as more extraction times gave insignificant increments in the yield of polysaccharides. In this study, the extraction yield optimized based on previous results, which is 3.27 ± 0.18% of dry basis, was quite high. 

In order to ensure the purity of the extracted polysaccharides, free proteins should be eliminated. Based on a conducted Bradford test, it was found that all extracts contained less than 0.1% protein (data not shown), regardless of the concentration of ethanol used. In this study, it was proven that samples extracted with a more dilute ethanol (75%) gave the least protein content, as higher concentrations would precipitate the protein. Free protein in the polysaccharides can be removed by several means, for example using the Sevag method [26], whereby the protein will became gel and precipitate in the Sevag reagent, which was composed of chloroform and *n*-butanol (4:1, v/v). However, in this study, proteins were naturally denatured due to heating of sample at 60 °C during the evaporation process. Denatured proteins will be precipitated and later removed through centrifugation [19]. This technique is much cheaper and safer for food application as less chemicals are involved.

### 2.2. Molecular Weight and Fractionation

**Figure 1:**
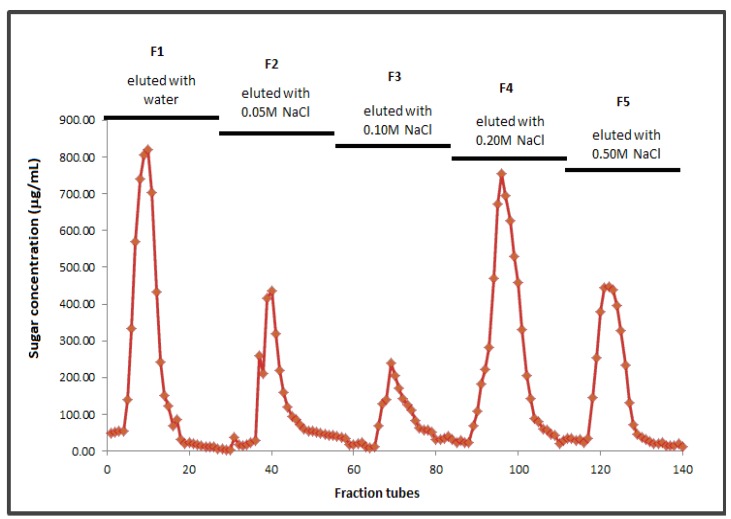
Anion-exchange chromatogram of BSCP (F1: Neutral polysaccharides–eluted with water; F2–F5: Acidic polysaccharide-eluted by salt gradient elution).

Standard calibration curve for series of T-dextrans gave a regression coefficient, R^2^ = 0.9959 with the equation y = −0.393x + 8.6256. Water soluble bamboo shoot crude polysaccharides (BSCP) was found to have a molecular weight of approximately 7.49 × 10^3^ Da. It was then purified and separated into five main sub-fractions, namely F1, F2, F3, F4 and F5. Fraction profiles are shown in Figure 1.

Sub-fraction F1 was neutral polysaccharides as it was eluted with water. Other fractions (F2–F5) were acidic polysaccharides, as they were eluted with an increasing concentrations of NaCl from 0.05 to 0.50 M through anion-exchange chromatography [27]. Since salt solutions can elute acidic polysaccharides from cellulose columns by anion-exchange, the response to NaCl gradients therefore indicated the acidity of F2 to F5 [28].

Molecular weights of all purified fractions (F1 to F5) were 1,550.96, 1,471.63, 1,685.78, 1,691.61 and 1,551.67 Da, respectively. It was found that the neutral polysaccharides, F1 had the lowest molecular weight as compared to other fractions. The molecular weight of polysaccharide is usually related to certain biological activities. Higher activity in biological complement system of polysaccharides was noted with the increase in the molecular weight of acidic polysaccharides [29]. According to Gullon *et al.* [30], the purity of fractions does not play any significant role in fermentation. Therefore, sub-fractions obtained through the fractionation process were not included as a carbon source for bacterial fermentation.

### 2.3. Structure of BSCP

#### Fourier Transform Infra-Red (FTIR)

**Figure 2:**
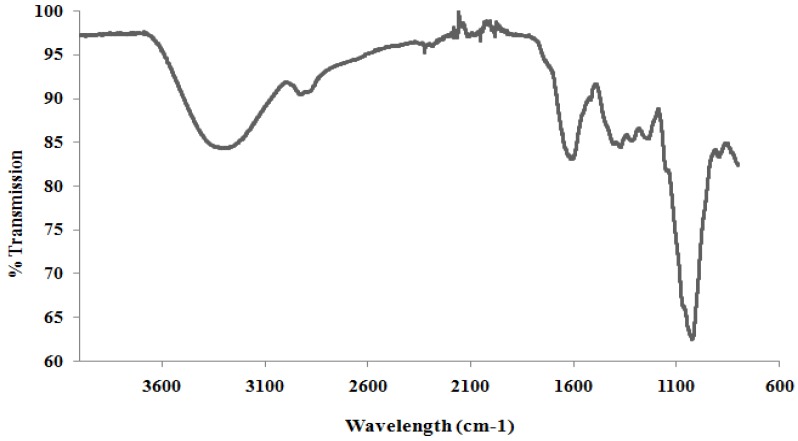
FTIR spectra for BSCP.

FTIR spectra for BSCP is shown in Figure 2. Broad peaks in the 950–1,200 cm^−1^ range indicate polysaccharide as a major component in the extract. This band is dominated by contributions from heavy atoms C-C and C-O stretching vibration in pyranoid rings [31,32,33]. For bands that lie between 1,200 and 1,500 cm^−1^, they are caused mainly by CH deformation vibrations and COH bending vibrations. Other peaks at around 2,920 cm^−1^ indicate C-H stretching as absorption peaks of sugar and a very broad peak around 3,400 cm^−1^ is assigned to the hydroxyl stretching vibration modes [31,33,34].

In carbohydrate analysis using IR spectra, α and β conformers can be clearly distinguished within the anomeric region–vibrational bands since the α and β-configuration are well separated in the 950 to 750 cm^−1^ region, where 870–840 cm^−1^ corresponds to α conformers, while the β conformers lie around 890 cm^−1^[31,35].

The band near to 894 cm^−1^ was reported as a β-glucosidic bond and therefore a weaker band found near to this region (891 cm^−1^ in BSCP) was believed to indicate the presence of β-glucan. Other bands and shoulders assigned as β-glucan were found near 1,372, 1,322 and 1,047 cm^−1^[15,36]. However, there are also traces of α-configuration such as C-C stretching and C-H bonding of α-glucose and α-D-glucose [31,37] components in the extract of BSCP shown as a very weak band near 833 to 853 cm^−1^. The peak at 463 cm^−1^ indicates the presence of α-1,3-glucan, while the bands at 1420 cm^−1^ (was an almost pure CH_2_ group vibration) and 1,240 cm^−1^ were derived from the associated and free bending vibrations of the hydroxyl group [31,34].

### 2.4. Digestibility of BSCP

Development of prebiotics has focused on the non-digestibility of oligosaccharides [38] to ensure they reach the colon and preferably persist throughout the large intestine such that benefits are apparent distally [5]. As a result, they can effectively stimulate certain indigenous bacteria resident in the gut rather than introducing exogenous species to the host [39]. 

**Figure 3:**
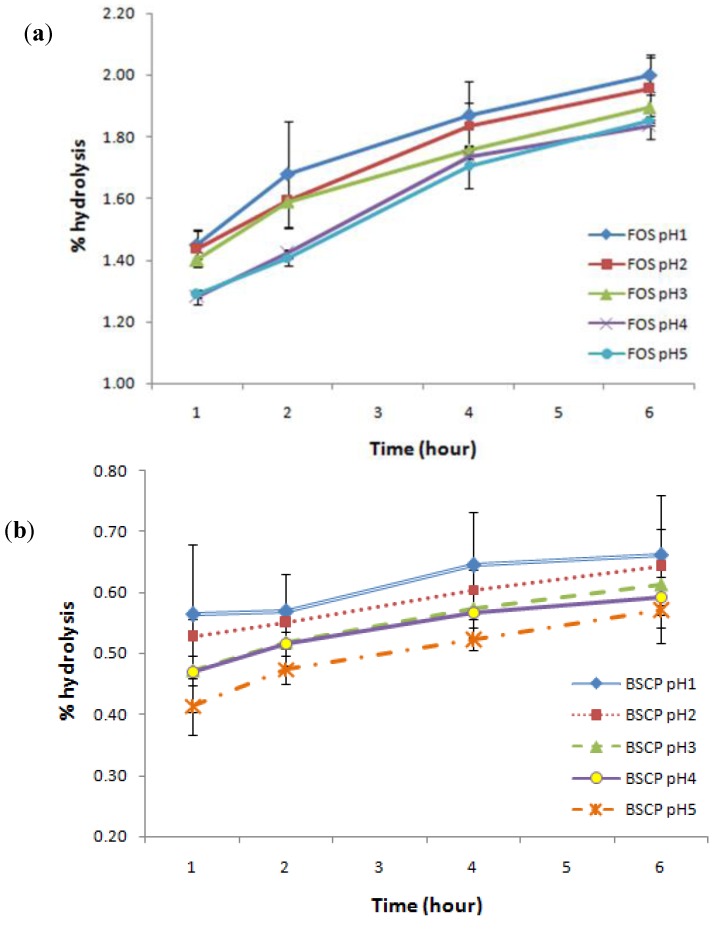
Degree of hydrolysis for both (**a**) FOS and (**b**) BSCP in artificial human gastric juice.

In this study, BSCP was found to be resistant towards artificial human gastric juice as compared to FOS. Surprisingly, the non-digestibility of BSCP was noted to be more than 99% at the lowest pH tested (pH 1). It also showed small differences in the degree of hydrolysis (Figure 3) within the incubation time (0 to 6 h) at all pH values tested (0.41 ± 0.05 to 0.66 ± 0.10%), as compared to FOS which ranges from 1.45 ± 0.05 to 2.00 ± 0.06%. From the data, it can be said that BSCP was less susceptible towards gastric juice, regardless of the incubation time, while hydrolysis was seen to continuously increase in the case of FOS. However, food is usually retained in the human stomach for about 2 h [38]. The results gathered gave a good indication that BSCP can be regarded as a potential prebiotic, as it meets prebiotic characteristics, which need it to be non-digestible. It was comparable to other oligosaccharides studied such as kojioligosaccharides, which are 100% resistant to artificial gastric acid [40] and showed better resistance than gluco-oligosaccharide produced by *Gluconobacter oxydans* NCIMB 4943 (98.4%) [41].

In this study, BSCP was found to have lower digestibility as compared to FOS, a commercial and well-known prebiotic. This means that BSCP is able to reach the colon safely without severe degradation by the human gastric juice as compared to FOS. BSCP stands a good chance to be further evaluated as a potential prebiotic as it meets the first characteristic of prebiotics [42]. Basically, the degree of digestibility reflects the susceptibility of the compound towards gastric juice, thus allowing the remaining compound to reach the colon intact and serve the probiotic bacteria. In the case of another example of commercial prebiotic, a galactooligosaccharide (GOS), more than 90% was reported to arrive into the colon [43]. Because of that, non-digestibility has been regarded as one of the prerequisites of any prebiotic effect of dietary ingredients.

### 2.5. Growth of Bacteria

Two bifidobacterial and one lactobacilli strains were chosen as probiotics in this study, while *Salmonella* sp. was used as a potential pathogenic control of prebiotic activity. All strains tested in this study showed a positive growth towards the supplementation of crude polysaccharides extracted from the shoots of *Gigantochloa levis *(BSCP) (Table 1). The colour of fermented broth was noticed to change from colourless to cloudy and deep yellow in all tubes, indicating polysaccharide fermentation by the bacterium. 

BSCP were found to have significant effect on the growth of probiotics, comparable to FOS at all concentrations tested. All probiotics showed an outstanding growth rate of about 10.49 to 15.94 times of its original cell density after 24 h of fermentation using BSCP as their carbon source, rather than FOS which indicated slower growth rates of about 10.46 to 14.54 (Table 2). Viability of probiotics in BSCP supplemented media stably showed a positive increase, even after 48 h, with that of *B. longum* found to be the highest. The complexity of BSCP structure was believed to demonstrate these effects as longer time is needed for the bacterium to digest the polymer and eventually supplying them with excess substrate to ensure their existence [44].

**Table 1:** Cell density of selected probiotics and S*almonella* sp. over fermentation time (at 600 nm).

Bacterial strains	Carbon source	Concentration (ppm)	Fermentation time (h)			
			0	24	48	72
***B. animalis***	Control	-	0.1163 ± 0.01 ^a^	0.8548 ± 0.03 ^e^	0.7772 ± 0.06 ^c^	0.5226 ± 0.12 ^c^
	FOS	250	0.1186 ± 0.01 ^a^	1.3933 ± 0.05 ^d^	1.4844 ± 0.19 ^b^	1.3919 ± 0.11 ^a^
	FOS	500	0.1107 ± 0.02 ^a^	1.7201 ± 0.26 ^bcd^	1.6216 ± 0.08 ^b^	1.5814 ± 0.08 ^a^
	FOS	750	0.1055 ± 0.02 ^a^	1.6335 ± 0.14 ^cd^	1.5093 ± 0.03 ^b^	1.0356 ± 0.18 ^b^
	FOS	1000	0.1127 ± 0.02 ^a^	1.6553 ± 0.12 ^bcd^	1.4308 ± 0.02 ^b^	1.4209 ± 0.17 ^a^
	BSCP	250	0.1209 ± 0.03 ^a^	1.8616 ± 0.08 ^abc^	1.9139 ± 0.19 ^a^	1.6740 ± 0.22^a^
	BSCP	500	0.1196 ± 0.03 ^a^	1.8667 ± 0.28 ^abc^	1.9268 ± 0.06 ^a^	1.6500 ± 0.11 ^a^
	BSCP	750	0.1205 ± 0.00 ^a^	2.0061 ± 0.27 ^ab^	1.9997 ± 0.25 ^a^	1.5276 ± 0.29 ^a^
	BSCP	1000	0.1227 ± 0.01 ^a^	2.0782 ± 0.22 ^a^	2.0477 ± 0.19 ^a^	1.7059 ± 0.10 ^a^
***B. longum***	Control	-	0.1085 ± 0.01 ^a^	0.6053 ± 0.10 ^c^	0.6367 ± 0.10 ^b^	0.4488 ± 0.11 ^b^
	FOS	250	0.1003 ± 0.01 ^a^	1.2930 ± 0.10 ^b^	1.4268 ± 0.05 ^a^	1.4867 ± 0.02 ^a^
	FOS	500	0.1020 ± 0.01 ^a^	1.2845 ± 0.06 ^b^	1.4655 ± 0.17 ^a^	1.4824 ± 0.10 ^a^
	FOS	750	0.1149 ± 0.02 ^a^	1.3171 ± 0.04 ^b^	1.4740 ± 0.12 ^a^	1.5403 ± 0.06 ^a^
	FOS	1000	0.1124 ± 0.02 ^a^	1.3381 ± 0.21 ^b^	1.5075 ± 0.14 ^a^	1.4695 ± 0.24 ^a^
	BSCP	250	0.1242 ± 0.01 ^a^	1.4576 ± 0.10 ^ab^	1.6077 ± 0.15 ^a^	1.5711 ± 0.07 ^a^
	BSCP	500	0.1249 ± 0.03 ^a^	1.4348 ± 0.22 ^ab^	1.6350 ± 0.18 ^a^	1.6075 ± 0.07 ^a^
	BSCP	750	0.1139 ± 0.02 ^a^	1.6637 ± 0.14 ^a^	1.6677 ± 0.05 ^a^	1.6142 ± 0.06 ^a^
	BSCP	1000	0.1166 ± 0.01 ^a^	1.7139 ± 0.24 ^a^	1.7139 ± 0.24 ^a^	1.5802 ± 0.17 ^a^
***L. acidophilus***	Control	-	0.1223 ± 0.01 ^a^	1.0444 ± 0.11 ^c^	1.0379 ± 0.05 ^c^	0.7057 ± 0.15 ^b^
	FOS	250	0.1187 ± 0.01 ^a^	1.5589 ± 0.10 ^b^	1.7159 ± 0.05 ^ab^	1.6722 ± 0.20 ^a^
	FOS	500	0.1267 ± 0.01 ^a^	1.5841 ± 0.01 ^b^	1.7705 ± 0.07 ^ab^	1.7743 ± 0.11 ^a^
	FOS	750	0.1153 ± 0.01 ^a^	1.5849 ± 0.09 ^b^	1.7459 ± 0.09 ^ab^	1.7240 ± 0.09 ^a^
	FOS	1000	0.1269 ± 0.01 ^a^	1.5965 ± 0.09 ^b^	1.6824 ± 0.05 ^b^	1.7611 ± 0.03 ^a^
	BSCP	250	0.1252 ± 0.01 ^a^	1.8167 ± 0.18 ^c^	1.9436 ± 0.23 ^ab^	1.8569 ± 0.07 ^a^
	BSCP	500	0.1294 ± 0.01 ^a^	1.8540 ± 0.13 ^c^	1.9501 ± 0.24 ^ab^	1.8629 ± 0.06 ^a^
	BSCP	750	0.1235 ± 0.01 ^a^	1.8733 ± 0.11 ^c^	1.9822 ± 0.16 ^a^	1.8650 ± 0.10 ^a^
	BSCP	1000	0.1293 ± 0.02 ^a^	1.8912 ± 0.19 ^c^	2.0053 ± 0.21 ^a^	1.8393 ± 0.13 ^a^
***Salmonella***	Control	-	0.1162 ± 0.03 ^a^	0.9632 ± 0.05 ^c^	0.9789 ± 0.13 ^b^	0.8351 ± 0.11 ^b^
	FOS	250	0.1172 ± 0.01 ^a^	1.3456 ± 0.06 ^ab^	1.4051 ± 0.22 ^a^	1.3943 ± 0.13 ^a^
	FOS	500	0.1147 ± 0.01 ^a^	1.1879 ± 0.10 ^b^	1.3427 ± 0.20 ^a^	1.4029 ± 0.15 ^a^
	FOS	750	0.1244 ± 0.01 ^a^	1.4754 ± 0.10 ^a^	1.4883 ± 0.18 ^a^	1.5029 ± 0.17 ^a^
	FOS	1000	0.1229 ± 0.01 ^a^	1.3560 ± 0.13 ^ab^	1.5154 ± 0.14 ^a^	1.5431 ± 0.15 ^a^
	BSCP	250	0.1201 ± 0.01 ^a^	1.4394 ± 0.15 ^a^	1.5205 ± 0.17 ^a^	1.3993 ± 0.19 ^a^
	BSCP	500	0.1187 ± 0.01 ^a^	1.4571 ± 0.09 ^a^	1.4273 ± 0.17 ^a^	1.3831 ± 0.18 ^a^
	BSCP	750	0.1229 ± 0.02 ^a^	1.4805 ± 0.14 ^a^	1.5354 ± 0.14 ^a^	1.5177 ± 0.14 ^a^
	BSCP	1000	0.1167 ± 0.02 ^a^	1.4372 ± 0.16 ^a^	1.5872 ± 0.21 ^a^	1.5849 ± 0.20 ^a^

* Note : Means with the same small letter (within each fermentation time and bacterial strains) were not significantly different at (p £ 0.05) according to Duncan’s Multiple Range test.

**Table 2:** The growth rate* of selected probiotics and *Salmonella *over fermentation time as supplemented with different concentration of FOS and BSCP.

Bacterial strains	Carbon Source	0–24 h	24–48 h	48–72 h
*B. animalis*	Control	6.35	−0.09	−0.33
	FOS 250 ppm	10.75	0.07	−0.06
	FOS 500 ppm	14.54	−0.06	−0.02
	FOS 750 ppm	14.48	−0.08	−0.31
	FOS 1000 ppm	13.69	−0.14	−0.01
	BSCP 250 ppm	14.40	0.03	−0.13
	BSCP 500 ppm	14.61	0.03	−0.14
	BSCP 750 ppm	15.65	0.00	−0.24
	BSCP 1000 ppm	15.94	−0.01	−0.17
*B. longum*	Control	4.58	0.05	−0.30
	FOS 250 ppm	11.89	0.10	0.04
	FOS 500 ppm	11.59	0.14	0.01
	FOS 750 ppm	10.46	0.12	0.04
	FOS 1000 ppm	10.90	0.13	−0.03
	BSCP 250 ppm	10.74	0.10	−0.02
	BSCP 500 ppm	10.49	0.14	−0.02
	BSCP 750 ppm	13.61	0.00	−0.03
	BSCP 1000 ppm	13.70	0.00	−0.08
*L. acidophilus*	Control	7.54	−0.01	−0.32
	FOS 250 ppm	12.13	0.10	−0.03
	FOS 500 ppm	11.50	0.12	0.00
	FOS 750 ppm	12.75	0.10	−0.01
	FOS 1000 ppm	11.58	0.05	0.05
	BSCP 250 ppm	13.51	0.07	−0.04
	BSCP 500 ppm	13.33	0.05	−0.04
	BSCP 750 ppm	14.17	0.06	−0.06
	BSCP 1000 ppm	13.63	0.06	−0.08
*Salmonella*	Control	7.29	0.02	−0.15
	FOS 250 ppm	10.48	0.04	−0.01
	FOS 500 ppm	9.36	0.13	0.04
	FOS 750 ppm	10.86	0.01	0.01
	FOS 1000 ppm	10.03	0.12	0.02
	BSCP 250 ppm	10.99	0.06	−0.08
	BSCP 500 ppm	11.28	−0.02	−0.03
	BSCP 750 ppm	11.05	0.04	−0.01
	BSCP 1000 ppm	11.32	0.10	0.00

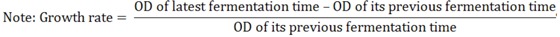

At the same time, a gradual reduction in the growth rate after 24 h of fermentation time was noted in *B. animalis* supplemented with FOS at a concentration of 500–1,000 ppm. Up to 72 h of fermentation, most of the carbon sources supplemented to the bacterium were being utilized and become limiting for the bacterium to survive. This was indicated through the reduction of measured cellular densities [45]. These demonstrated the ability of BSCP to be metabolized by the bacteria, supporting the theory it has potential prebiotic properties.

It is difficult to compare the effectiveness of prebiotic activity of BSCP to other compounds as different concentrations of compounds were used in different studies. In addition, the choice of probiotics and pathogen were also different. Synytsya *et al.* [15] studied the prebiotic activity of glucans extracted from *Pleurotus ostreatus* and *Pleurotus eryngii* without comparing them to any commercial prebiotic. A study done by Vergara *et al.* [11] proved the prebiotic potential of fermented cashew apple (*Anacardium occidentale L*.) juice by comparing its activity against sucrose, glucose and fructose. Again, no dose-response relationship was noted as a fixed concentration was used. The same condition was noted with the study conducted by Wichienchot *et al.* [17]. No dose-response relationship was declared for the determination of prebiotic activity of oligosaccharides from pitaya flesh against inulin. The gap in the study of prebiotic activity could be filled by establishing a standard method comprising of standard bacterial strains and commercial prebiotic. However, it might be difficult to implement due to enormous number of bacterial strains and prebiotics available. 

Multiplication of bacteria over time caused an increase in the amount of short chains fatty acids (acetate, propionate, lactate and butyrate) produced, which eventually gradually lowered the pH of the fermented broth along with the increase of bacteria (data not shown). The decrease in pH values after incubation with BSCP suggests that the probiotics were able to utilize BSCP as their carbon source. This again being an indicator that BSCP possesses good potential for exploitation as a prebiotic.

The definition of a prebiotic requires it to selectively stimulate beneficial intestinal bacteria and at the same time be able to suppress the growth of pathogenic bacteria. In this study, it was found that both BSCP and FOS could support the growth of *Salmonella* spp. rather than suppressing its growth at earlier stages. However, its growth rate was less than those of probiotics at all fermentation time points. In this case, the differences can only be seen after 48 h of fermentation time, where the growth of *Salmonella* sp. was noted to decline, while most of probiotics to continuously grew up to 72 h.

**Figure 4:**
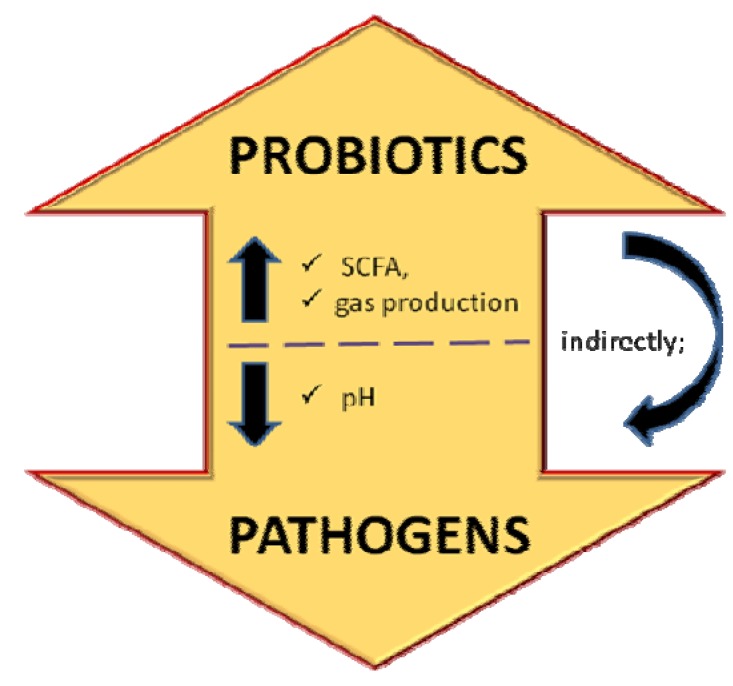
Prebiotics mechanism in manipulating intestinal microbiota.

The question whether prebiotics are selective towards beneficial intestinal bacteria has been debated by most researchers, as other study also shows a virtual utilization of prebiotics by pathogenic bacteria [44,46,47]. The possibility of these prebiotics are utilized by pathogenic bacteria *in vivo* is highly probable based on conducted *in vitro* studies. However, Teitelbaum and Walker [47] suggested that the effect of prebiotics was actually indirect, because it is the changes in the gastrointestinal microbiota compositions (bifidobacteria, lactobacilli, as well as the histolyticum subgroup; bacteroides and clostridia) that give rise to the prebiotics effect. Furthermore, different people harbour different bacterial species and the composition of the microbiota can be affected by a variety of other factors such as diet, disease, drugs, antibiotic, age and others [48]. Metabolism of prebiotics can be regarded as simultaneous 3-in 1-actions which can be simplified in Figure 4.

## 3. Experimental

### 3.1. Extraction of Polysaccharides

#### 3.1.1. Bamboo Shoots

The shoots used in this study were *Gigantochloa levis* (locally known as *Buluh beting*). It was purchased from a farmer in Johol, Negeri Sembilan. Sample were peeled off and dried in a vacuum-oven at 50 °C until a constant weight was obtained. They were then ground and sieved using a No. 400 sieve in order to standardize the particle size.

#### 3.1.2. Chemicals

Absolute ethanol was purchased from HmbG Chemicals, Malaysia. Fructooligosaccharides (FOS) was obtained from BENEO Orafti, Belgium.

#### 3.1.3. Extraction

Samples were stirred continuously in 80% ethanol for 5 h using a magnetic stirrer with heating on a ceramic heating plate (C-MAG HS 7 IKAMAG®), followed by hot water bath extraction at 90 °C for 3 h. Four layers of cotton cheese cloth were used for filtration purposes. Filtrate was then concentrated to 1/50 of its original volume using a rotary evaporator at 60 °C. Insoluble materials were removed by centrifugation at 8,000 rpm for 10 min, before subjected to dialysis (T3 membrane, Fisherbrand; MW cutoff = 12 kDa) in distilled water for 24 h at 4 °C. Ethanol precipitation was carried out to isolate the polysaccharides present in the dialyzed sample. The precipitate was then oven-dried at 50 °C to a constant weight. Protein content was quantified by using Bradford’s method [49] using bovine serum albumin (BSA) as the standard as to check the level of purity. One mL of 20 µg/mL sample was mixed with 2 mL of dye and incubated for 10 min prior to measurement at 595 nm using UV-VIS spectrophotometer.

### 3.2. Molecular Weight Estimation

The molecular weight of crude polysaccharides was determined using a gel chromatography technique using High Performance Liquid Chromatography system, equipped with a Waters 600 Controller and Refractive Index detector (Waters 410) with a manual injector. Standard T-series dextrans with molecular weights of 25, 50, 80, 150, 180, 220, 270 and 410 kDa were used at 1% (w/v) concentration. Standard T-series dextrans and glucose (20 µL) were passed through a Zorbax GF-450 column using isocratic mode at a flow rate of 1 mL/min with deionized water as a mobile phase. The column temperature was set at ambient. The elution times were plotted against the logarithm of their respective molecular weights.

### 3.3. Fractionation

Purification of BSCP was done following the method proposed by Chen *et al.* [50] and Bao *et al.* [51]. Dried BSCP (0.5 g) was diluted in distilled water (250 mL) and applied to a DEAE-Sepharose™ Fast Flow column. BSCP was eluted with distilled H_2_O followed stepwise by 0.05, 0.1, 0.2 and 0.5 M NaCl solutions. Each fraction (8 mL) was collected at a flow rate of 2.0 mL/min and analyzed for total carbohydrate content by the phenol-sulphuric acid method using method proposed by Dubois [52]. Fractions were then pooled together into a few large portions depending on their sugar content (Fraction 1: Tubes 5 to 15; Fraction 2: Tubes 36 to 46; Fraction 3: Tubes 67 to 77; Fraction 4: Tubes 84 to 94 and Fraction 5: Tubes 103 to 111) before being analyzed for its molecular weight.

### 3.4. Structural Elucidation of Polysaccharides

#### Fourier Transform Infra-Red (FTIR) Spectroscopy

FTIR spectra (spectral region range from 4,000–600 cm^−1^ with resolution 2 cm^−1^ and 128 scans) of the extracted water soluble polysaccharides from *Gigantochloa levis* was directly measured on FTIR spectrophotometer using a Nicolet iN10/iZ10 with a DTGS detector and a Golden Gate single reflection Diamond HATR sampling accessories (Thermo Scientific, USA). It is used to investigate the vibrations of molecules and polar bonds between the different atoms. Types of monosaccharide, glycosidic bonds and functional groups can also be determined using FTIR spectroscopy [37].

### 3.5. Resistance towards Acid Digestibility

Non-digestibility of oligosaccharides is defined as the ability to resist digestive processes which includes resistance towards gastric acidity, hydrolysis by mammalian enzymes and gastrointestinal absorption [38]. Digestibility of crude polysaccharides extracted from bamboo shoot was tested by calculating the degree of its hydrolysis when subjected to artificial human gastric juice. Analysis was conducted based on study done by [17]. Artificial human gastric juice was mimicked by using hydrochloric acid buffer containing (in g/L): NaCl, 8; KCl, 0.2; Na_2_HPO_4_·2H_2_O, 8.25; NaHPO_4_, 14.35; CaCl_2_·2H_2_O, 0.1; MgCl_2_·6H_2_O, 0.18. This buffer was adjusted to pH 1 to 5 using 5 M HCl. This buffer (5 mL at each pH) was added to sample solution (1% w/v, 5 mL) and incubated in a water bath (37 ± 1 °C) for 6 h. Sample (1 mL) was taken periodically at 0, 0.5, 1, 2, 4 and 6 h and tested for reducing sugar content using the dinitrosalicyclic acid (DNS) assay [53] and also total sugar content using the phenol-sulphuric acid method [52]. In this experiment, FOS was used as a control. Percentage of hydrolysis of sample was calculated based on reducing sugar released and total sugar content of the sample as below:

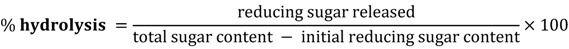

where reducing sugar released is the difference between its final and initial content.

### 3.6. Media and Microorganisms

#### 3.6.1. Media

Medium used for fermentation was modified from formulation proposed by [54]. The pH was standardized to pH 6.5 ± 0.1 using phosphate buffer. Fructooligosaccharides and BSCP were added into broth medium at a concentration of 250, 500, 750 and 1000 ppm prior to sterilization. Control medium containsed only water. The medium was then inoculated with *B. animalis* ATCC 1053, *B. longum* BB 536 and *L. acidophilus *ATCC 4356 under aseptic condition and incubated anaerobically using Anaerocult A GasPac system (Merck, Darmstadt, Germany) at 37 °C. *S. choleraesuis* JCM 6977 on the other hand was incubated aerobically at 37 °C. Sampling for pH and Optical Density (OD) were done at 0, 24, 48 and 72 h. All measurements were done in duplicates.

#### 3.6.2. Microorganisms

All bacterial strains were obtained from the stock culture collection of Microbiology Laboratory (Lab202) (Faculty of Biotechnology and Biomolecular Sciences, UPM, Malaysia). Prior to analysis, Gram staining was performed in order to ensure all cultures were pure and not contaminated during storage. Cultures stored were maintained at −20 °C in 20% (v/v) glycerol. Probiotic bacteria was activated by growing it in MRS broth supplemented with 0.05% L-cystein for 48 h under anaerobic conditions using Anaerocult A GasPac system (Merck, Darmstadt, Germany) at 37 °C. Active cells were then transferred twice at 1% inoculation concentration (v/v) in MRS broth medium followed by incubation for 24 h prior to use.

### 3.7. Prebiotic Activity

#### 3.7.1. pH Measurement

pH of fermented broth over time interval was determined using a pH meter (Mettler Toledo, USA), which was calibrated using buffers of pH 4.0, pH 7.0 and pH 10.0 prior to analysis.

#### 3.7.2. Determination of Optical Density (OD)

Optical density was tested at every 0, 24, 48 and 72 h of incubation period. These will reflect the survivality of bacteria in different added concentration of carbon source. Duplicate samples were measured spectrophotometrically using a UV-Visible spectrophotometer at 600 nm. Results were expressed as unit of cell densities.

### 3.8. Statistical Analysis

Data gathered were analyzed by using one-way ANOVA. Duncan’s Multiple Range Test was applied to determine the significant difference between the incubation times at *p* ≤ 0.05. All tests were performed using the Statistical Analysis System software version 6.12.

## 4. Conclusions

BSCP was found to potentially support the growth of probiotics better than FOS. This gave a good indication that BSCP could be exploited further as a prebiotic, as not all carbohydrates could exert similar beneficial effects as commercial prebiotics do [44]. BSCP can be considered a long chain polysaccharide with an average molecular weight of 7,000 Da. It contains β-glycosidic bonds which lead to its high degree of non-digestibility (more than 99%). In conclusion, bamboo shoots represent a promising source of natural prebiotics. More tests, including human trials should be considered.
